# Comparison of kidney and hepatic outcomes among sodium-glucose cotransporter-2 inhibitors: a retrospective study using multiple propensity scores

**DOI:** 10.1186/s40780-024-00378-2

**Published:** 2024-09-17

**Authors:** Kazuya Hiura, Chinami Suzuki, Junichi Kubo, Haruka Goto, Shigo Takatori, Kiyomi Ishida, Yuki Tanaka, Akifumi Mizutani, Yuki Yamashita, Chiho Kurumazuka, Akihiko Takagi, Ryu Kobayashi, Akio Shibanami

**Affiliations:** 1 Department of Clinical Pharmaceutics, Faculty of Pharmaceutical Sciences, Hokkaido University of Science, 7-15-4-1 Maeda, Teine, Sapporo, Hokkaido 006-8590 Japan; 2 Hospital Pharmacy, Obihiro Kosei General Hospital, Obihiro, 080-0024 Japan; 3 Hospital Pharmacy, Asahikawa Kosei General Hospital, Asahikawa, 078-8211 Japan; 4 Hospital Pharmacy, Sapporo Kosei General Hospital, Sapporo, 060-0033 Japan; 5 Hospital Pharmacy, Abashiri Kosei General Hospital, Abashiri, 093-0076 Japan; 6 Hospital Pharmacy, Engaru Kosei General Hospital, Engaru, 099-0404 Japan

**Keywords:** SGLT2i, Renoprotective, Hepatoprotective, IPTW

## Abstract

**Background:**

Sodium-glucose cotransporter-2 inhibitors (SGLT2i) have been reported to have effects beyond lowering blood glucose levels, with certain SGLT2i expanding their indications to chronic kidney disease and chronic heart failure. We focused on the hepatoprotective and renoprotective effects of six SGLT2i and assessed whether the effects were unique to each drug or common class effects, in addition to whether the renal and hepatoprotective effects vary based on renal and hepatic status.

**Methods:**

Patients with diabetes (ipragliflozin: 837, empagliflozin: 850, canagliflozin: 922, dapagliflozin: 590, tofogliflozin: 288, and luseogliflozin: 193) who initiated SGLT2i treatment and were monitored for one year were included. The propensity score (PS) was calculated using patient backgrounds (age, sex, height, weight, body mass index [BMI], disease duration, concomitant diabetes medications, underlying conditions, glycated hemoglobin [HbA1c], estimated glomerular filtration rate [eGFR], aspartate aminotransferase [AST], alanine aminotransferase [ALT], high-density lipoprotein [HDL], low-density lipoprotein [LDL], and triglyceride [TG] levels) as covariates. Additionally, the inverse probability of treatment weighting (IPTW) approach was used to compare liver and renal function test values.

**Results:**

Pre- and 12-month post-treatment comparisons demonstrated a significant reduction in hepatic function (AST and ALT) and an increase in renal function (eCcr and eGFR) for all SGLT2i. Comparison of differences between pre- and 12-month post-treatment using the IPTW approach demonstrated no significant differences in AST, ALT, and eGFR levels between SGLT2i. At 12 months post-treatment, 67 patients were classified as having a more severe CKD than those at pre-treatment, representing only 1.8% of all patients (67/3,680). Similarly, 107 patients with AST and 147 patients with ALT were classified as having progressed to a more severe grade than at pre-treatment, representing only 2.9 and 4.0%, respectively.

**Conclusions:**

Renoprotective and hepatoprotective effects are class effects of SGLT2i, and their effects are thought to be independent of kidney or liver status.

**Supplementary Information:**

The online version contains supplementary material available at 10.1186/s40780-024-00378-2.

## Background

Sodium-glucose cotransporter-2 inhibitors (SGLT2i) are drugs that lower blood glucose levels without insulin action by inhibiting glucose reabsorption in the proximal tubule, thereby facilitating urinary glucose excretion [1]. In Japan, six SGLT2i (ipragliflozin [IGZ], empagliflozin [EGZ], canagliflozin [CGZ], dapagliflozin [DGZ], tofogliflozin [TGZ], and luseogliflozin [LGZ]) have been approved for the treatment of diabetes. Initially, SGLT2i were anticipated to lower blood glucose levels; reduce body weight, blood pressure, and uric acid levels; and enhance lipids, insulin secretory reserve, and insulin resistance [2–6]. Additionally, renoprotective [7–11] and hepatoprotective effects [12, 13] have recently attracted attention. DGZ is indicated for chronic kidney disease (CKD), and CGZ for CKD complicated by type 2 diabetes in Japan. Despite studies indicating the hepatoprotective effects of SGLT2i in patients with type 2 diabetes [12, 13], their use in liver diseases has not yet been explored. Currently, our understanding of whether all six SGLT2i exhibit renoprotective and hepatoprotective effects, and variations in the effects among the six drugs, in addition to whether the renal and hepatoprotective effects differ based on renal and hepatic status, is limited.

In this study, patients who initiated treatment with SGLT2i were monitored for one year to assess alterations in renal and liver function tests. The inverse probability of treatment weighting (IPTW) approach [14] was used for drug comparisons, in which the propensity score (PS) was calculated using the patient background, and the inverse of the PS was used as the weight.

## Methods

### Target patients

Patients with diabetes at JA Hokkaido Koseiren hospitals (Asahikawa Koseiren, Sapporo Koseiren, Obihiro Koseiren, Abashiri Koseiren, Engaru Koseiren, and Kutchan Koseiren) for whom treatment with SGLT2i was initiated from April 2014 to March 2020 and who were monitored for 12 months were eligible for the study. As a control, patients who were not taking SGLT2i, for whom treatment with dipeptidyl peptidase-4 inhibitor (DPP-4i) was initiated, and who were monitored for 12 months were also studied. DPP4i is the most prescribed drug for diabetes in Japan. Patients for whom treatment with drugs that exhibit renoprotective and hepatoprotective effects was initiated were excluded. The drugs excluded for their renoprotective effects were the three drugs reported to be effective for diabetic nephropathy, namely angiotensin-converting enzyme inhibitors, angiotensin II receptor blockers, and mineralocorticoid receptor antagonists [15–23]. Additionally, the drugs excluded for their hepatoprotective effects were nine drugs identified through a keyword search in Japanese package inserts for “improvement in liver function” and “improvement in hyperammonemia” (including ursodeoxycholic acid, methylmethionine sulphonium chloride, polyene phosphatidylcholine, taurine, tiopronin, diisopropylamine dichloroacetate, monoammonium glycyrrhizinate/glycine/aminoacetic acid/dl-methionine, lactulose, and rifaximin).

### Survey variables

#### Patient background

We assessed patient background, such as age, sex, height, weight, body mass index (BMI), diabetes duration, concomitant use of hypoglycemic drugs (DPP-4i, sulfonylurea [SU], α-glucosidase inhibitor [α-GI], glinide [GL], biguanide [BG], and thiazolidinediones [TZ]), underlying diseases (liver disease, renal disease, heart disease, cerebrovascular disease, hypertension, and dyslipidemia) and laboratory values (glycated hemoglobin [HbA1c], estimated glomerular filtration rate [eGFR], aspartate aminotransferase [AST], alanine aminotransferase [ALT], high-density lipoprotein [HDL], low-density lipoprotein [LDL], and triglycerides [TG]). In all cases, the data were obtained before the initiation of SGLT2i or DPP4i treatment.

#### Alterations in laboratory values associated with renal and hepatic function

We assessed renal function (eGFR) and hepatic function (AST and ALT) laboratory values pre- and 6 and 12 months post-SGLT2i treatment initiation. Additionally, alterations in laboratory values (ΔeGFR, ΔAST, and ΔALT) were calculated using the following formula as an assessment index of renoprotective and hepatoprotective effects. Calculation formula: (test values after 12 months of SGLT2i treatment)−(test values before SGLT2i treatment).

#### Statistical analysis

Statistical analyses were conducted using JMP® Pro 17 (SAS Institute Inc., Cary, NC, USA). The Mann–Whitney *U*-test was used to assess two related groups, whereas the Kruskal–Wallis test was used to assess multiple groups. The IPTW approach was used to compare ΔeGFR, ΔAST, and ΔALT values to adjust for factors that may affect renoprotective and hepatoprotective effects. To calculate the PS required for the IPTW approach, 25 patient background variables were used as covariates. Using this approach, we intended to perform adjustment among SGLT2i and did not use DPP4i patient background variables. *P* values < 0.05 were considered statistically significant.

### Ethical considerations

This study was approved by the ethics committees at each hospital (Asahikawa Kosei [approval number: 2020056], Sapporo Kosei [approval number: 554], Obihiro Kosei [approval number: 2020–089], Abashiri Kosei [approval number: 202012], Engaru Kosei [approval number: 2020–13], and Kutchan Kosei [approval number: R2-3]). In this study, we used only existing data, without acquiring written or oral consent from the patients. Therefore, we disclosed data regarding the study on the web or posted them in the hospital (or both), and guaranteed the opportunity for all patients to decline participation.

## Results

### Patient background

There were 3,680 cases (IGZ: 837, EGZ: 850, CGZ: 922, DGZ: 590, TGZ: 288, and LGZ: 193) (Table 1). There were 7,172 control DPP4i cases. To assess covariate balance in the SGLT2i cases, a standardized difference score (Std diff) was calculated. |Std diff|< 0.1 was considered a minor difference [24]. |Std diff| was > 0.1 for age, weight, BMI, diabetes duration, concomitant medications (α-GI), heart disease, cerebrovascular disease, hypertension, dyslipidemia, HbA1c, and HDL-C (Table 2). This indicates an uneven distribution of SGLT2i patient backgrounds.

**Table 1:** Patient background

	IGZ (*n* = 837)	EGZ (*n* = 850)	CGZ (*n* = 922)	DGZ (*n* = 590)	TGZ (*n* = 288)	LGZ (*n* = 193)	DPP4i (*n* = 7,172)
Age, year	55.2 ± 14.9	60.2 ± 13.9	58.0 ± 15.6	52.2 ± 17.0	46.8 ± 14.6	49.7 ± 17.7	69.5 ± 12.1
Sex, males/females	579/258	579/271	614/308	410/180	164/124	134/59	4,390/2,782
Height, cm	162.3 ± 9.8	161.5 ± 10.5	160.8 ± 11.0	162.1 ± 10.1	161.2 ± 10.8	161.7 ± 11.0	159.9 ± 10.0
Weight, kg	72.4 ± 17.1	70.9 ± 17.3	70.8 ± 18.0	72.9 ± 17.5	76.5 ± 17.4	73.2 ± 17.4	60.3 ± 13.5
BMI	27.5 ± 6.2	27.2 ± 6.1	27.4 ± 7.1	27.8 ± 6.7	29.6 ± 6.7	28.2 ± 6.7	23.5 ± 4.2
Diabetes duration, year	0.7 ± 1.3	1.4 ± 2.4	1.1 ± 1.4	1.5 ± 2.6	1.3 ± 1.6	1.5 ± 2.2	1.5 ± 2.4
DPP4i, + / −	534/303	465/385	595/327	352/238	173/115	106/87	-
SU, + / −	186/651	173/677	212/710	89/501	49/239	29/164	1,943/5,229
α-GI, + / −	112/725	81/769	66/856	41/549	10/278	8/185	515/6,657
GL, + / −	44/793	34/816	46/876	24/566	12/276	3/190	289/6,883
BG, + / −	337/500	344/506	315/607	226/364	99/189	84/109	1,700/5,472
TZ, + / −	56/781	67/783	42/880	34/556	10/278	12/181	130/7,042
Liver disease, + / −	78/759	119/731	64/858	57/533	20/268	11/182	3,016/4,156
Renal disease, + / −	66/771	99/751	70/852	49/541	33/255	11/182	2,719/4,453
Heart disease, + / −	174/663	210/640	174/748	106/484	58/230	13/180	2,732/4,440
Cerebrovascular disease, + / −	35/802	35/815	36/886	14/576	8/280	0/193	1,043/6,129
Hypertension, + / −	183/654	243/607	165/757	124/466	55/233	26/167	2,678/4,494
Dyslipidemia, + / −	228/609	281/569	193/729	164/426	51/237	26/167	2,421/4,751
HbA1c, %	8.3 ± 1.8	8.1 ± 1.7	7.8 ± 1.6	8.2 ± 1.7	8.4 ± 1.9	8.4 ± 2.0	8.0 ± 2.2
eGFR, mL/min/1.73m^2^	67.1 ± 29.7	64.4 ± 28.0	64.3 ± 28.7	61.5 ± 28.5	61.3 ± 33.2	56.8 ± 26.5	63.9 ± 27.6
AST, IU/L	30.4 ± 18.9	31.3 ± 17.1	31.1 ± 25.1	30.9 ± 18.1	30.7 ± 19.3	31.9 ± 16.9	32.7 ± 65.1
ALT, IU/L	34.8 ± 26.5	33.9 ± 22.2	34.0 ± 24.6	36.2 ± 26.9	36.6 ± 26.6	37.5 ± 24.9	31.1 ± 53.4
HDL-C, mg/dL	44.8 ± 17.5	48.4 ± 16.6	49.5 ± 16.7	49.0 ± 17.9	49.6 ± 17.7	47.0 ± 16.3	49.4 ± 15.8
LDL-C, mg/dL	116.4 ± 37.2	115.6 ± 37.4	114.9 ± 36.8	114.3 ± 34.9	120.5 ± 39.8	119 ± 34.9	109.5 ± 34.0
TG, mg/dL	166.7 ± 189.3	165.7 ± 123.2	158.5 ± 93.7	153.4 ± 81.1	180.5 ± 160.0	169.6 ± 92.6	154.1 ± 141.5

Data are expressed as the mean ± standard deviation (SD)

*IGZ* Ipragliflozin, *EGZ* Empagliflozin, *CGZ* Canagliflozin, *DGZ* Dapagliflozin, *TGZ* Tofogliflozin, *LGZ* Luseogliflozin, *BMI* Body mass index, *DPP-4i* Dipeptidyl peptidase-4 inhibitor, *SU* Sulfonylurea, *GL* Glinide, *α-GI* α-glucosidase inhibitor, *BG* Biguanide, *TZ* Thiazolidinediones, *HbA1c* Glycated hemoglobin, *eGFR* Estimated glomerular filtration rate, *AST* Aspartate aminotransferase, *ALT* Alanine aminotransferase, *HDL-C* High-density lipoprotein-cholesterol, *LDL-C* Low-density lipoprotein-cholesterol, *TG* Triglyceride

**Table 2:** Standardized difference scores pre- and post-adjustment in SGLT2i

	|Std diff|	Adjusted |Std diff|
Age	0.358	0.217
Sex	0.071	0.094
Height	0.059	0.040
Weight	0.137	0.017
BMI	0.139	0.014
Diabetes duration	0.152	0.118
DPP4i	0.083	0.026
SU	0.092	0.046
α-GI	0.218	0.041
GL	0.088	0.025
BG	0.072	0.050
TZ	0.062	0.048
Liver disease	0.096	0.050
Renal disease	0.080	0.023
Heart disease	0.116	0.023
Cerebrovascular disease	0.219	0.078
Hypertension	0.109	0.059
Dyslipidemia	0.167	0.027
HbA1c	0.123	0.009
eGFR	0.077	0.029
AST	0.016	0.013
ALT	0.051	0.013
HDL-C	0.110	0.018
LDL-C	0.063	0.053
TG	0.077	0.014

*Std diff* Standardized difference scores, *BMI* Body mass index, *DPP-4i* Dipeptidyl peptidase-4 inhibitor, *SU* Sulfonylurea, *GL* Glinide, *α-GI* α-glucosidase inhibitor, *BG* Biguanide, *TZ* Thiazolidinediones, *HbA1c* Glycated hemoglobin, *eGFR* Estimated glomerular filtration rate, *AST* Aspartate aminotransferase, *ALT* Alanine aminotransferase, *HDL-C* High-density lipoprotein-cholesterol, *LDL-C* Low-density lipoprotein-cholesterol, *TG* Triglyceride

### Renal function laboratory values

**Fig. 1:**
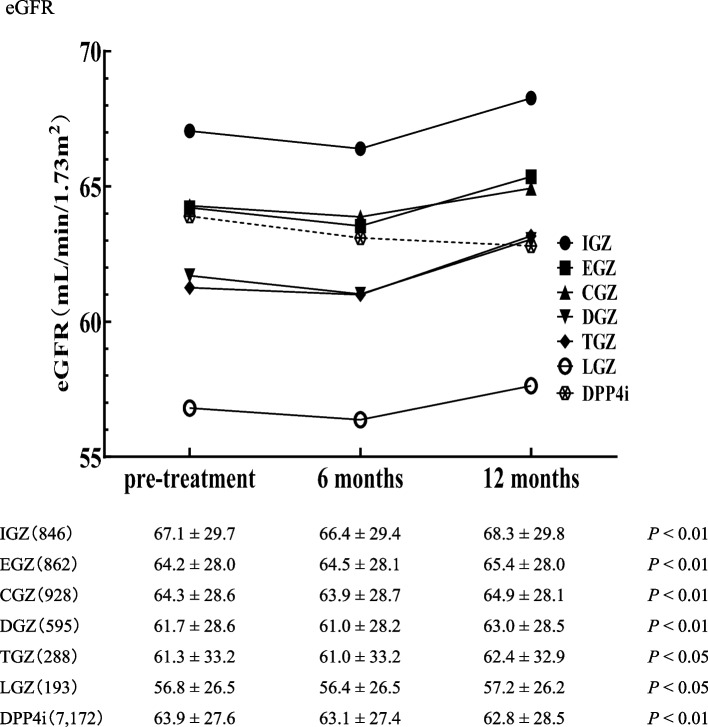
Alteration in eGFR levels pre-, 6, and 12 months post-SGLT2i or DPP4i treatment. Data are presented as the mean ± SD. The Mann–Whitney U-test was used to compare eGFR values pre- and 12 months post-SGLT2i or DPP4i treatment

There was a significant reduction in eGFR levels before treatment across all SGLT2i compared to 12 months post-treatment (Fig. 1). For all SGLT2i, there was a temporary reduction in eGFR levels at six months. There was a significant reduction in the DPP4i control.

Pre-treatment eGFR levels with SGLT2i were classified based on CKD severity as follows: G1 (eGFR ≥ 90: 583), G2 (90 > eGFR ≥ 30: 1,384), G3 (60 > eGFR ≥ 30: 1,272), and G4 (30 > eGFR: 441; Fig. 2). At 12 months post-treatment, 67 patients (G1: 37 and G2: 30) were classified as having a more severe CKD than those at pre-treatment, representing only 1.8% of all patients (67/3,680). The eGFR levels in 92.6% of the patients were either increased, unaltered, or reduced within the classification criteria.

**Fig. 2:**
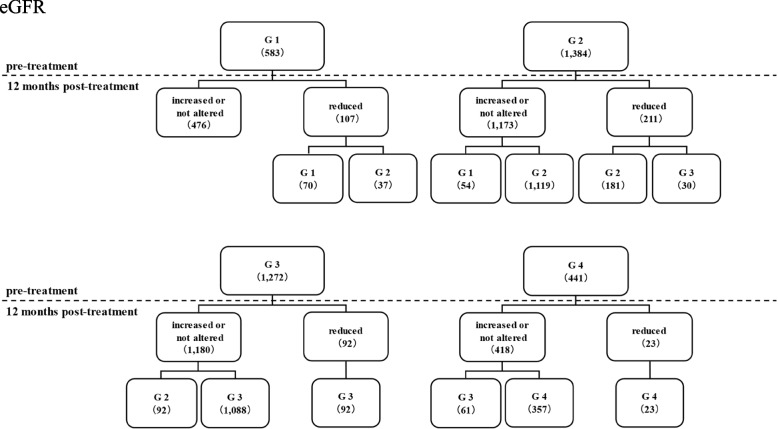
Classification of eGFR levels using reference values. eGFR pre- and 12 months post-SGLT2i treatment was classified as G1 (eGFR ≥ 90), G2 (90 > eGFR ≥ 30), G3 (60 > eGFR ≥ 30), and G4 (30 > eGFR) according to the severity classification of CKD

### Hepatic function laboratory values

**Fig. 3:**
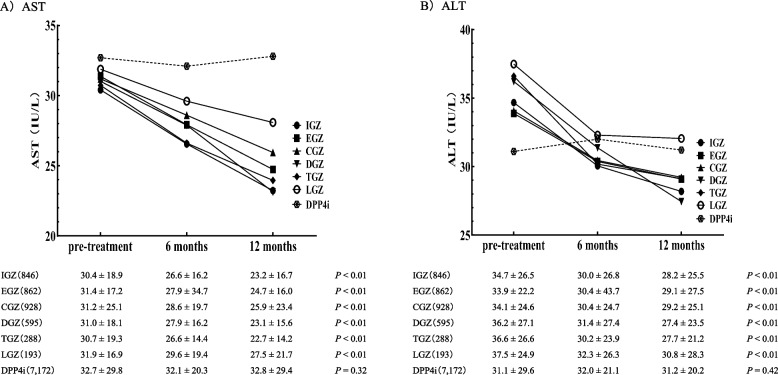
Alteration in AST and ALT levels pre-, 6, and 12 months post-SGLT2i or DPP4i treatment. Data are presented as the mean ± SD. The Mann–Whitney U-test was used to compare eGFR values pre- and 12 months post-SGLT2i or DPP4i treatment

There was a significant reduction in AST and ALT levels before treatment across all SGLT2i compared to 12 months post-treatment (Fig. 3). There were no significant alterations in AST and ALT in the DPP4i control.

**Fig. 4:**
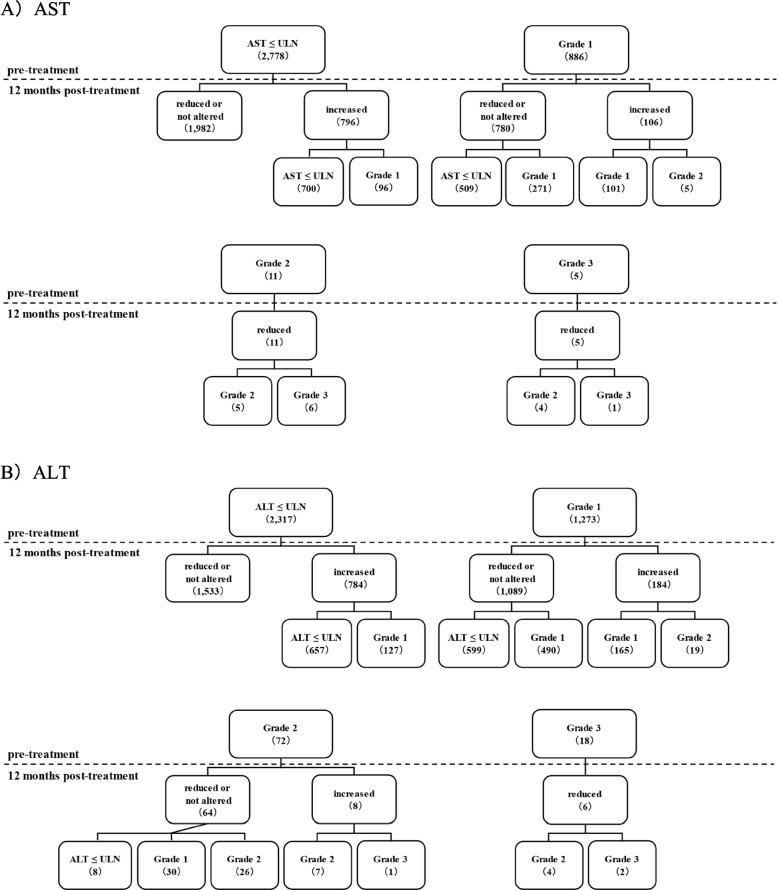
Classification of AST and ALT levels using reference values. AST or ALT pre- and 12 months post-SGLT2i treatment were classified as below ULN (AST: ≤ 38 IU/L, ALT: male ≤ 44, female ≤ 23), Grade 1 (38 < AST ≤ 114, male: 44 < ALT ≤ 132, female: 23 < ALT ≤ 69), Grade 2 (114 < AST ≤ 190, male: 132 < ALT ≤ 220, female: 69 < ALT ≤ 115), and Grade 3 (190 < AST ≤ 760, male: 220 < ALT ≤ 880, female: 115 < ALT ≤ 460) according to the severity classification of CTCAE ver5.0

Pre-treatment AST and ALT levels with SGLT2i were classified based on common terminology criteria for adverse events (CTCAE) ver5.0 as follows: below upper limit of normal (ULN; AST: ≤ 38, ALT: male ≤ 44, female ≤ 23), Grade 1 (ULN—ULN*3; 38 < AST ≤ 114, male: 44 < ALT ≤ 132, female: 23 < ALT ≤ 69), Grade 2 (ULN*3—ULN*5; 114 < AST ≤ 190, male: 132 < ALT ≤ 220, female: 69 < ALT ≤ 115), Grade 3 (ULN*5—ULN*20; 190 < AST ≤ 760, male: 220 < ALT ≤ 880, female: 115 < ALT ≤ 460; Fig. 4). The respective numbers of cases of AST and ALT were 2,778 and 2,317 for below ULN; 886 and 1,273 for Grade 1; 11 and 8 for Grade 3, and 5 and 6 for Grade 3.

In the below ULN AST group, 96.5% (2,682/2,778) of patients remained normal after 12 months, whereas 56.4% (509/902) of patients in the high AST group (38 <) returned to normal after 12 months. In the below ULN ALT group, 94.5% (2,190/2,317) of patients remained normal after 12 months, whereas 44.5% (607/1,363) of patients in the high ALT group (male: 44; female: 23) returned to normal after 12 months.

### Alterations in renal and hepatic function laboratory values

**Fig. 5:**
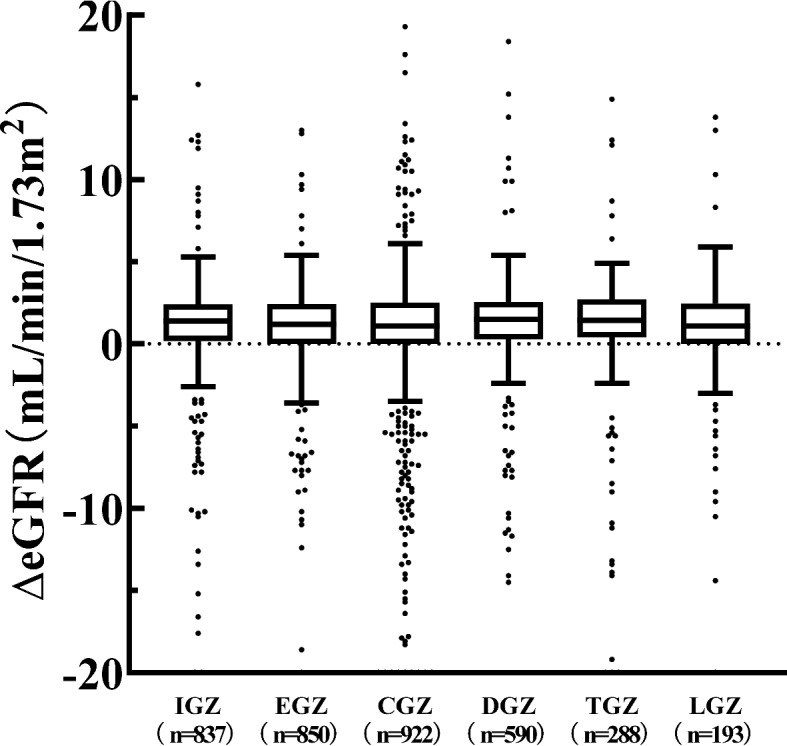
Box plots of ΔeGFR in pre- and post-SGLT2i treatment. The two ends of the whiskers represent the minimum and maximum values in the range of the first quartile + 1.5*interquartile range (IQR) to third quartile + 1.5*IQR. Data beyond the ends of the whiskers are plotted individually. Inbox bars represent the median for ΔeGFR of each group. ΔeGFR: (eGFR post 12 months of SGLT2i treatment)−(eGFR pre-SGLT2i treatment). IPTW was performed and tested using the Kruskal–Wallis test

**Fig. 6:**
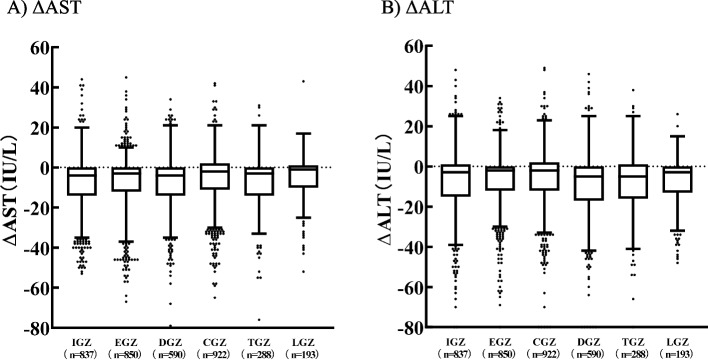
Box plots of ΔAST or ΔALT in pre- and post-SGLT2i treatment. The two ends of the whiskers represent the minimum and maximum values in the range of the first quartile + 1.5*interquartile range (IQR) to the third quartile + 1.5*IQR. Data beyond the ends of the whiskers are plotted individually. Inbox bars represent the median for ΔAST or ΔALT of each group. ΔAST: (AST post 12 months of SGLT2i treatment)−(AST pre-SGLT2i treatment). ΔALT: (ALT post 12 months of SGLT2i treatment)−(ALT pre-SGLT2i treatment). IPTW was performed and tested using the Kruskal–Wallis test

PS was calculated using 25 patient background variables as covariates (Table 1). Patient background was adjusted using the IPTW approach, with 1/PS used as a weight in the statistical analysis. Weight, BMI, concomitant medications (α-GI), diseases (heart disease, cerebrovascular disease, hypertension, and dyslipidemia), and HbA1c and HDL-C levels with |Std diff|≥ 0.1 could be adjusted to < 0.1. The |Std diff| adjusted for diabetes duration was 0.118, which was approximately 0.1. However, the |Std diff| for age was 0.217, even after adjustment, indicating a persistent distributional imbalance. No significant difference was observed in ΔeGFR, ΔAST, or ΔALT levels when comparing SGLT2i with homogenized patient backgrounds (Fig. 5, 6).

## Discussion

This study revealed that SGLT2i treatment maintained eGFR, AST, and ALT levels pre- and one year post-SGLT2i treatment. This indicates that all six SGLT2i exhibit renoprotective and hepatoprotective effects. Additionally, the IPTW approach was used to compare the alterations in eGFR, AST, and ALT levels pre- and one year post-treatment, and no significant differences were observed. These results indicate that renoprotective and hepatoprotective effects are common to SGLT2i. Furthermore, our data suggest that eGFR, AST, and ALT are enhanced regardless of liver or kidney status.

A comparison of SGLT2i using the receipt database with a focus on renal function indicated that the renoprotective effect of SGLT2i was a class effect [25]. This aligns with the results of this study, in which 25 variables of pre-treatment patient data were used to homogenize patient backgrounds through the IPTW approach. Sub-analyses of the dapagliflozin and prevention of adverse outcomes (DAPA)-CKD Trial (for DGZ) [26, 27] and the CREDENCE Trial (for CGZ) [8, 28] indicated renoprotective effects in patients with severely impaired renal function (eGFR < 30 mL/min/1.73 m^2^). In this study, renoprotective effects were observed at all CKD severity levels (G1 to G4), which we consider a novelty. Additionally, ΔeGFR was compared between SGLT2i for each CKD severity category, and no significant differences were observed (Additional file 1).

Additionally, an initial dip [29] (transient reduction in renal function post-administration, followed by an increase) was observed in all SGLT2i. The initial dip was observed in the early phase of treatment. In this study, the data at 6 months demonstrated a slight reduction in eGFR levels compared to that of the pre-treatment data. The CREDENCE Trial [8] reported a reduction in eGFR levels of 3.72 mL/min/1.73 m^2^ after three weeks of CGZ treatment. Compared with this report, the difference between the pre-treatment and 6-month follow-up was small, indicating that eGFR was in the process of recovery. This indicates that the renoprotective effects of SGLT2i should be assessed in the medium term (6–12 months).

Depending on the state of proteinuria and blood sugar control, diabetes causes a gradual decline in renal function [30]. It has also been reported that GFR declines at a rate of 0.36 mL/min/1.73 m^2^/year after the age of 40, even without renal disease [31]. Cases treated with DPP4i exhibited a significant decrease in eGFR. However, whether this was due to the progression of diabetes, aging, or other factors cannot be determined based on the data collected in this study. On the other hand, cases treated with SGLT2i exhibited a slight increase or maintenance of eGFR. While improvements in values are worth evaluating, we believe that maintaining stable kidney function without deterioration is crucial in diabetes treatment because diabetes causes a gradual decline in renal function.

Hepatoprotective effects were assessed based on alterations in the AST and ALT levels. AST and ALT levels decreased by 6.5 and 6.2 IU/L, respectively, after one year compared to the pre-treatment levels. Approximately 56.4% of patients with high pre-treatment AST levels and 44.5% of patients with high pre-treatment ALT levels achieved normal values. The pre-treatment AST and ALT grade classifications worsened after 12 months in only 108 and 147 cases, respectively. These results indicate that SGLT2i enhances and maintains liver function. Additionally, SGLT2i (six drugs) were divided into grades of AST and ALT levels to compare ΔAST and ΔALT, and no significant differences were observed between the drugs (Additional files 2 and 3). Fatty liver increases insulin resistance, which is associated with the development and severity of diabetes [32], and increased insulin resistance promotes further fat accumulation in the liver. SGLT2i improves hyperinsulinemia [33] and insulin resistance [34], which is thought to be involved in correcting fatty liver and improving and maintaining liver function. On the other hand, sitagliptin, classified as DPP-4i, has been reported to not improve fatty liver [35]. Blood insulin levels and insulin resistance could not be evaluated in this study. A positive correlation between insulin resistance and the TG/HDL ratio has been reported [36, 37]. An AST/ALT ratio ≤ 1 is an indicator of fatty liver due to overnutrition, in which ALT is predominantly elevated. Results with missing data but a significant decrease in the TG/HDL-Cho ratio and a positive correlation between ΔALT and the ΔTG/HDL-Cho ratio have been confirmed (Additional file 4 and 5). We thus infer that the improvement of hepatic function and insulin resistance is involved, although this is an indirect assessment. We believe that maintaining stable liver function without deterioration is crucial in diabetes treatment because reduced liver function (fatty liver) increases insulin resistance, which is associated diabetes progression. A Japanese study group of non-alcoholic fatty liver disease (NAFLD) [38] studied 1,365 cases of NAFLD and reported that the presence of diabetes was a risk factor for advanced fibrosis in non-alcoholic steatohepatitis (NASH). Additionally, according to the NASH study group of Japan’s Ministry of Health, Labour, and Welfare, the risk of death from hepatocellular carcinoma is the highest among malignant tumors in patients with diabetes [39]. Monitoring liver function is crucial for AST and ALT levels, liver fibrosis markers, and FibroScan results.

However, this study has limitations. Summarize the aforementioned as well. First, we could not evaluate blood insulin levels and insulin resistance. Second, we could not identify NASH or NAFLD and assess factors beyond AST, ALT levels. Third, diabetes has various complications, resulting in diverse patient backgrounds, but we used the IPTW approach to homogenize patient backgrounds to the maximum. In this study, we used 25 items for weighing, resulting in an average |Std diff| of 0.065, < 0.1. However, we failed to reduce the |Std diff| to < 0.1 for weight, BMI, duration of disease, and age. Despite this limitation, we believe that the IPTW approach is valuable for analyzing intricate clinical data. Finally, our data indicated that alterations in kidney- and liver-related laboratory values did not differ between SGLT2 inhibitors, but it was not possible to determine whether the mechanism of action was the same.

## Conclusions

Renoprotective and hepatoprotective effects are class effects of SGLT2i that are thought to be independent of kidney or liver status. This study lasted only one year. Because diabetes is a chronic disease, it is essential to assess the long-term persistence of its protective effects.

## Supplementary Information

Additional file 1.

Additional file 2.

Additional file 3.

Additional file 4.

Additional file 5.

## Data Availability

The datasets supporting the conclusions of this article are included within the article and its additional files.

## Abbreviations

SGLT2i: Sodium-glucose cotransporter-2 inhibitors
PS: Propensity score
HbA1c: Glycated hemoglobin
eCcr: Estimated creatinine clearance
eGFR: Estimated glomerular filtration rate
AST: Aspartate aminotransferase
ALT: Alanine aminotransferase
HDL-C: High-density lipoprotein-cholesterol
LDL-C: Low-density lipoprotein-cholesterol
TG: Triglyceride
IGZ: Ipragliflozin
EGZ: Empagliflozin
CGZ: Canagliflozin
DGZ: Dapagliflozin
TGZ: Tofogliflozin
LGZ: Luseogliflozin
CKD: Chronic kidney disease
IPTW: Inverse probability of treatment weighting
BMI: Body mass index
DPP-4i: Dipeptidyl peptidase-4 inhibitor
SU: Sulfonylurea
GL: Glinide
α-GI: α-Glucosidase Inhibitor
BG: Biguanide
TZ: Thiazolidinediones
Std diff: Standardized difference score
CTCAE: Common terminology criteria for adverse events
ULN: Upper limit of normal
NAFLD: Non-alcoholic fatty liver disease
NASH: Non-alcoholic steatohepatitis
IQR: Interquartile range
IPTW: Inverse probability of treatment weighting
DAPA: Dapagliflozin and prevention of adverse outcomes
