# Supplementary material for: Comparison of kidney and hepatic outcomes among sodium-glucose cotransporter-2 inhibitors: a retrospective study using multiple propensity scores
Source: J Pharm Health Care Sci. 2024 Sep 17;10:57. doi: 10.1186/s40780-024-00378-2 (PMC11407018; doi:10.1186/s40780-024-00378-2)
Supplement: Supplementary file 1 — Additional file 1. [file 40780_2024_378_MOESM1_ESM.pdf]

Additional file 1

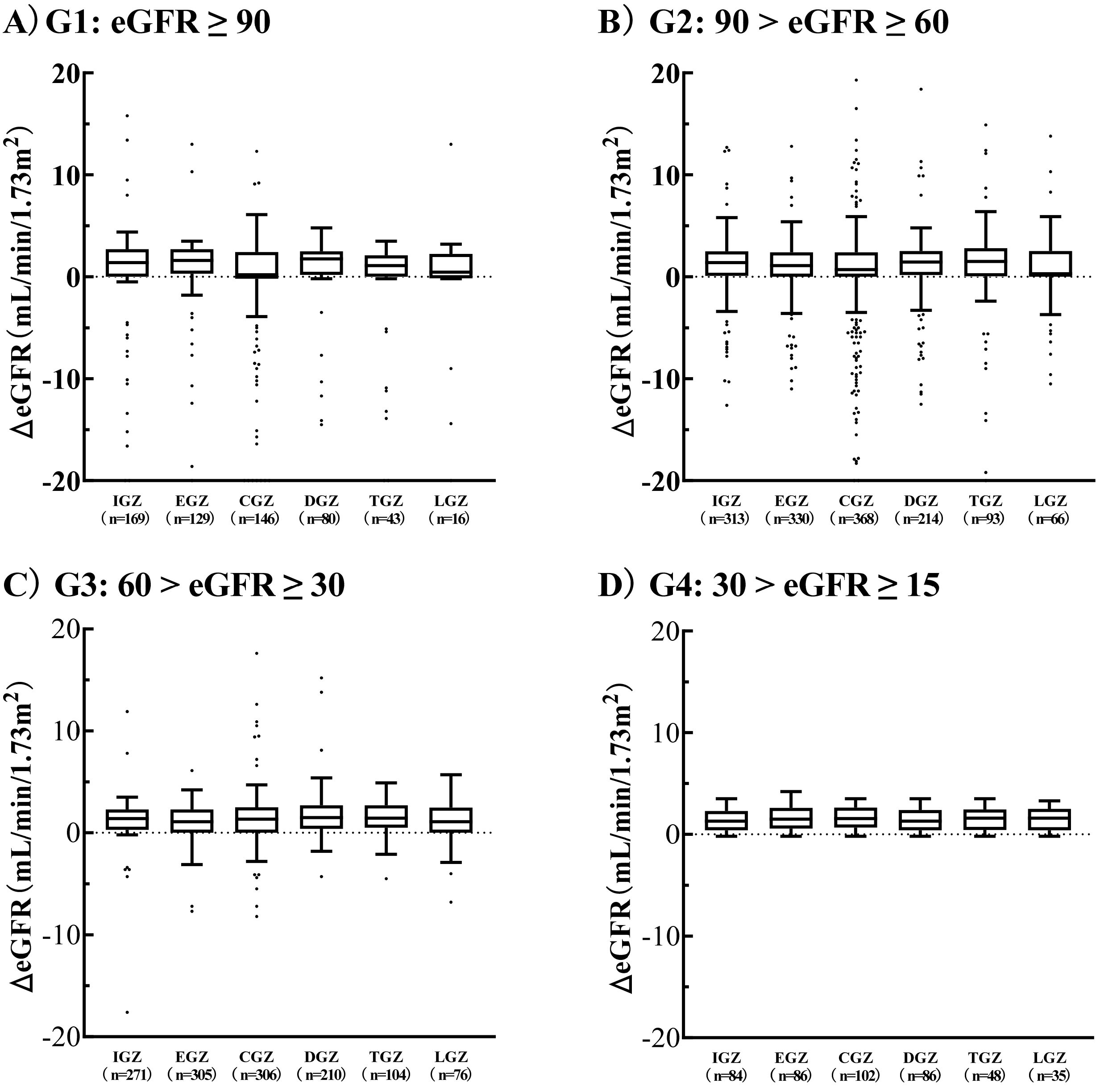

Box plot of ΔeGFR pre- and post-SGLT2i treatment through CKD severity classification.

The two ends of the whiskers represent the minimum and maximum values in the range of the first quartile +1.5*interquartile range (IQR) to the third quartile +1.5*IQR. Data beyond the ends of the

whiskers are plotted individually. Inbox bars represent the median for ΔeGFR of each group. ΔeGFR: (eGFR post 12 months of SGLT2i treatment) - (eGFR pre-SGLT2i treatment). IPTW was performed and tested using the Kruskal-Wallis test.
