# Supplementary material for: Comparison of kidney and hepatic outcomes among sodium-glucose cotransporter-2 inhibitors: a retrospective study using multiple propensity scores
Source: J Pharm Health Care Sci. 2024 Sep 17;10:57. doi: 10.1186/s40780-024-00378-2 (PMC11407018; doi:10.1186/s40780-024-00378-2)
Supplement: Supplementary file 2 — Additional file 2. [file 40780_2024_378_MOESM2_ESM.pdf]

Additional file 2

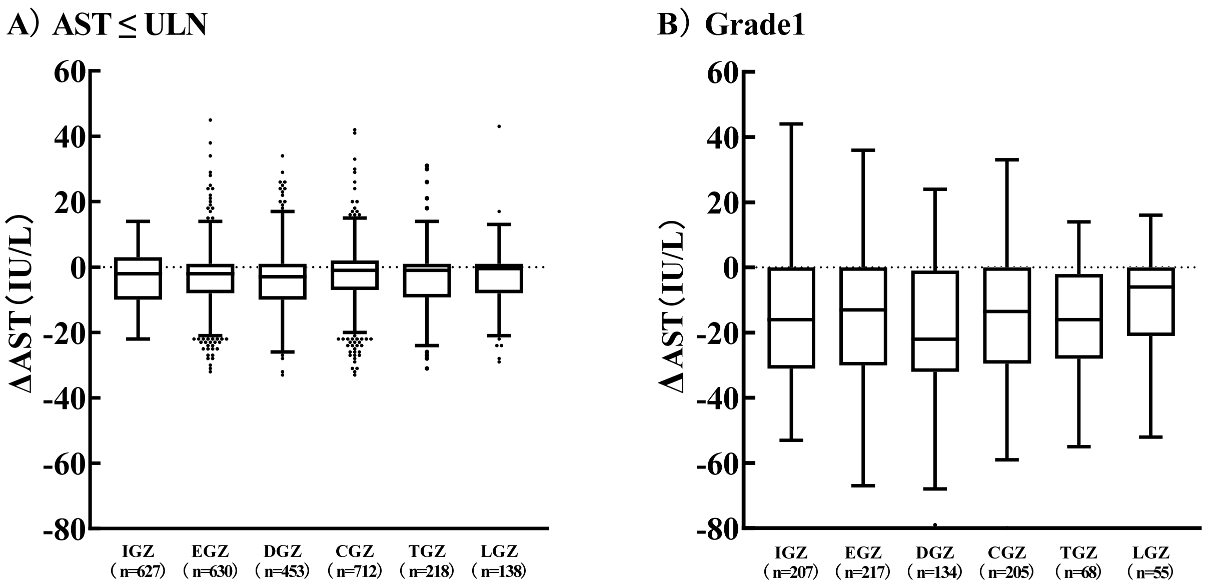

Box plot of ΔAST in pre- and post-SGLT2i treatment through CTCAE classification.

The two ends of the whiskers represent the minimum and maximum values in the range of the first quartile +1.5*interquartile range (IQR) to the third quartile +1.5*IQR. Data beyond the ends of the whiskers are plotted individually. Inbox bars represent the median for ΔAST of each group. ΔAST: (AST post 12 months of SGLT2i treatment) - (AST pre-SGLT2i treatment). IPTW was performed and tested using the Kruskal-Wallis test.
