# Supplementary material for: Comparison of kidney and hepatic outcomes among sodium-glucose cotransporter-2 inhibitors: a retrospective study using multiple propensity scores
Source: J Pharm Health Care Sci. 2024 Sep 17;10:57. doi: 10.1186/s40780-024-00378-2 (PMC11407018; doi:10.1186/s40780-024-00378-2)
Supplement: Supplementary file 5 — Additional file 5. [file 40780_2024_378_MOESM5_ESM.pdf]

Additional file 5

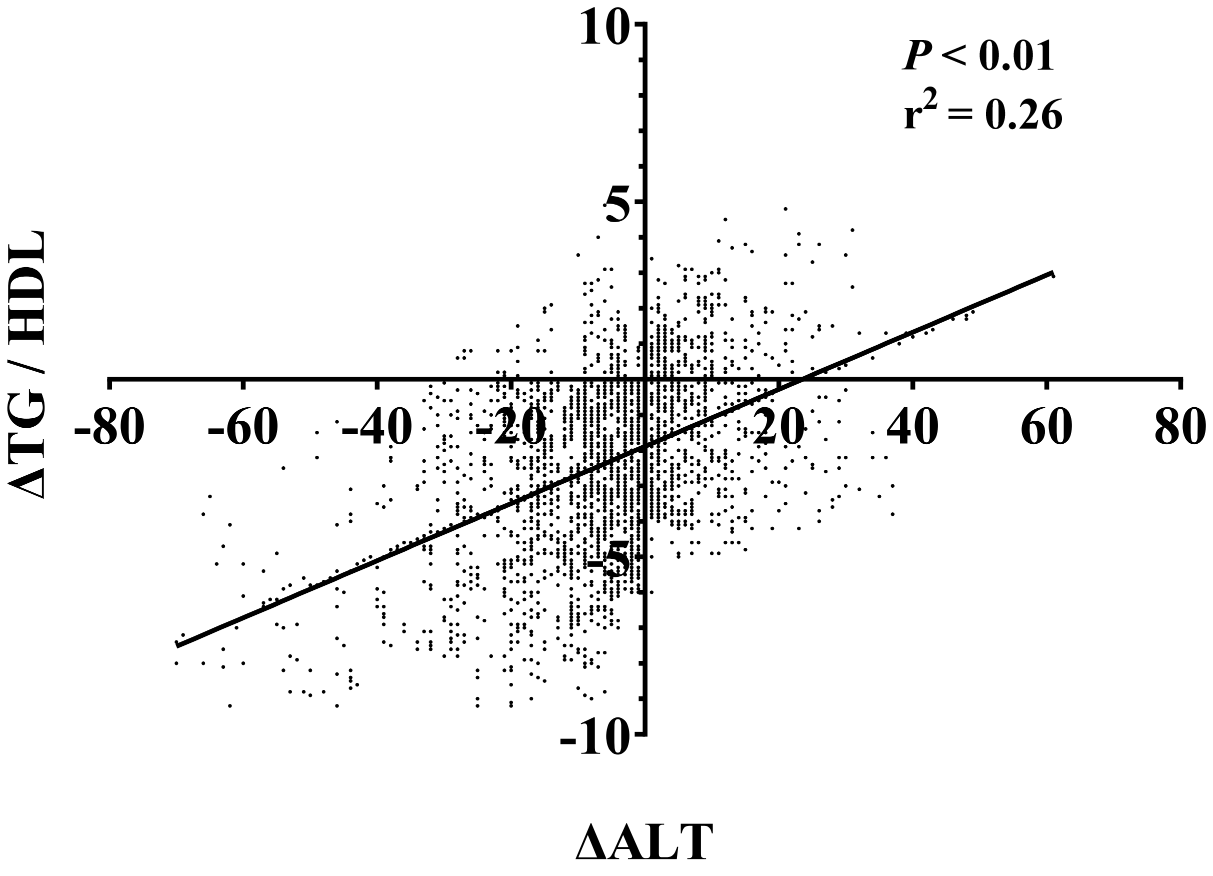

Correlation between ΔALT and ΔTG/HDL-C.

The x-axis shows ΔALT (ALT post 12 months of SGLT2i treatment) - (ALT pre-SGLT2i treatment), and the y-axis shows ΔTG/HDL-C (ΔTG / HDL-C post 12 months of SGLT2i treatment) - (ΔTG / HDL-C pre-SGLT2i treatment). Tested using the Pearson's correlation coefficient test.
